# Clinical patterns and their prevalence among adult population with back pain: A community-based cross-sectional study in rural Gadchiroli, India

**DOI:** 10.7189/jogh.11.12004

**Published:** 2021-11-27

**Authors:** Shekhar Y Bhojraj, Anand A Bang, Mahesh Deshmukh, Sameer Kalkotwar, Vinay R Joshi, Tushar Yarmal, Raghu Varma, Yogeshwar V Kalkonde, Abhay T Bang

**Affiliations:** 1 Spine Foundation, Mumbai, Maharashtra, India; 2 Society for Education, Action and Research in Community Health (SEARCH), Gadchiroli, Maharashtra, India; 3 Hinduja Hospital and Research Center, Mumbai, Maharashtra, India; 4 Naraindas Morbai Budhrani Trust, Mumbai, Maharashtra, India

## Abstract

**Background:**

Evaluating clinical patterns and their prevalence of back pain, a common problem in rural areas, can help develop treatment strategies to address this leading cause of disability.

**Methods:**

We conducted a population-based study in rural Gadchiroli, India. In this, two-phase study, trained surveyors conducted a door to door survey (Phase 1) to identify individuals with pain in the back and extremities in two villages randomly selected using pre-defined criteria. Those with pain were evaluated by a team of spine surgeons and rheumatologists to diagnose clinical conditions among these patients (Phase 2).

**Results:**

Of the 2535 eligible adults, 2259 (89%) were screened, 1247 (55%) reported pain in back and limb and were referred to the specialist clinic. Out of the 906 (73%) participants who attended the clinics, 783 (89%) had back/neck pain. The point prevalence of back/neck pain among adults was 49% (95% confidence interval (CI) = 49%-51%), non-specific low back pain 45% (95% CI = 43.4%-47.5%); non-specific neck pain 21% (95% CI = 18.9-22.4), radiculopathy 12 (95% CI = 10.4-13.1), myelopathy 0.4 (95% CI = 0.1-0.7) and other serious spinal disorders 0.2 (95% CI 0.048-0.45). The prevalence of non-specific back/neck pain and radiculopathy was higher among females.

**Conclusions:**

Non-specific back and neck pain are the commonest diagnoses among those with pain in the back and extremities, followed by radiculopathy. Serious disorders are rare. Given the high prevalence of non-specific back and neck pain, community health workers and physicians working in rural areas need to be trained systematically to manage these conditions.

Musculoskeletal disorders are the commonest cause of disability world over [1-3]. Back pain and other spinal disorders account for a significant number of patients with musculoskeletal disorders. One sixth of the world’s population lives in India and more than two-third of population of India lives in rural areas [4]. However, population-level information on the prevalence of the clinical patterns among adults with back pain is limited. A few studies have been conducted in rural India to assess the prevalence of back pain and other musculoskeletal complaints but these have been from the peri-urban and affluent areas [5,6]. Conducting such studies is important as a large part of the rural population of India is dependent upon heavy manual labour for earning livelihood. In such a setting, back pain and other spinal disorders are likely to be more prevalent and impose substantial economic and health burden on the individual, the family and the society at large.

This study was undertaken after the people of Gadchiroli district, one of the most underdeveloped districts of India, reported musculoskeletal pain as one of their most important health problems. It is a part of a programme to study musculoskeletal disorders in rural Gadchiroli in order to develop appropriate solutions. The objective of this study was to evaluate the clinical patterns among patients with back pain and estimate their prevalence in adults in rural Gadchiroli.

## METHODS

### Study setting

The study was conducted in Gadchiroli district of the Maharashtra state of India. The total population of the district is 1 071 795 as per the National Census conducted in 2011 [7]. The main source of livelihood is paddy cultivation. Health care is provided primarily through the public health system comprising one large district hospital, 13 smaller rural hospitals, 45 primary health care centers (PHCs) and 376 health subcenters (HSC). In addition, a few non-government organizations, traditional healers, unregistered doctors, private registered practitioners also provide health care.

Society for Education, Action and Research in Community Health (SEARCH) is a non-governmental organization working in this district since 1986 and has a field practice area of 86 villages spread in 3 blocks (revenue divisions of the district). In these villages, community health workers (CHWs) regularly collect population-based information as part of the demographic surveillance system and provide health care for selected ailments. The study was conducted by SEARCH in collaboration with the Spine Foundation, Mumbai a non-profit organisation to promote spine care in India and the rheumatology section, Hinduja Hospital, a large tertiary care hospital in Mumbai, India.

### Study design and sample

This study was a population-based, cross-sectional, interview-based survey of the prevalence of back pain in rural Gadchiroli. The sample size required for the primary study was calculated considering the anticipated point prevalence of non-specific low back pain in the adults (≥20 years of age) at 15%. To determine prevalence, with 95% confidence, precision of 0.02, design effect of 25% and non-response rate of 15% a minimum sample of 1800 adults was needed. Considering the average population of adults in villages in the field practice area of SEARCH at about 1000, 2 villages were needed to be included in the study.

The villages for the study were selected from 86 villages in the field practice area of SEARCH. The inclusion criteria for villages were a) presence of residential male and female CHW of SEARCH in the village to ensure complete data collection, b) adult (≥20 years) population ≥1000, c) villages should be more than 5 km away from Gadchiroli town and d) the village should not have a hospital or PHC. The aim was to include typical Indian villages of medium size, not too close to an urban area, and where a house to house survey was feasible by the CHWs of SEARCH. Villages with larger adult population (>2000) were excluded. Seven villages met the eligibility criteria forming the sampling frame of the study. From these seven villages, two villages were randomly selected. The two selected villages, Mudza and Bamhani were 7 and 12 km from the district headquarter respectively and 20 km from each other. Both had perennial roads. A health sub center manned by an Auxiliary Nurse and Midwife (ANM) providing immunization and maternity services was present in both the villages. Both the villages were agrarian with farming as the primary source of livelihood. All the resident adults ≥20 years of age of these two villages as recorded in the population register of SEARCH were eligible to be included the study and were recruited through household survey by the CHW.

### Data collection

Adults were screened for pain in the back and extremities (PBE) by trained surveyors using a validated form in a door-to-door survey. Patients with PBE were evaluated in the specialty clinic conducted in the village using a standardized form. This form was pilot tested in a spine and a rheumatology clinic in the city of Mumbai as well as in the rural clinic of SEARCH and modified appropriately before using in the survey. The method of interviewing and clinical examination as well as the clinical and diagnostic definitions and criteria were standardized and the spine surgeons who used these underwent a one-day training workshop.

### Data

The data for the prevalence of pain in back and extremities were collected from 1 January 2010 to 25 January 2010 by the trained CHWs of SEARCH. The CHWs conducted a door to door survey and after taking an informed consent, administered the questionnaire in a face-to-face interview with the eligible male and female participants. All patients with PBE were referred to the village-based clinic staffed by a medical team which included spine surgeons, senior rheumatologists, physiotherapist, occupational therapist and a psychologist.

The clinics were organized in respective villages approximately 15 days after the completion of the data collection for the prevalence study to reduce the time period between identification of symptoms (pain in back and extremities) by the CHW and the subsequent examination by the clinician as well as to minimize any possible inconsistencies in the findings of the CHWs and the clinician attributed to the time elapsed between the two visits.

#### Diagnosis and classification

The diagnoses were made based on clinical evaluation. Standard operational definitions were used for various clinical diagnoses. Neck pain was defined as midline pain in the region between lower border of occipital bone to the shoulders, upper back pain as midline pain between shoulders to the costal arch and, low back pain was defined as midline pain in the area between the costal arch and the tail bone. Back pain was defined as any pain in neck, upper or lower back. The pain was defined as ‘non-specific’ if there were no red flags (supplementary panel) which indicated presence of serious underlying disorder. Radiculopathy was diagnosed if individuals had pain along the distribution of the nerve root with or without neurological deficits in the distribution of the nerve root. Myelopathy was diagnosed if the participant had constellation of symptoms and signs suggestive of spinal cord pathology (eg, presence of exaggerated reflexes, Babinski reflex, spasticity, weakness, a sensory level or gait abnormalities). Laboratory and imaging investigations were performed as necessary and were provided free of charge to the patients. Teams also made home visits to the participants who were in the village but were unable to visit the clinic due to disability or age. The patients were managed according to the standard management plan. All the participants were provided with appropriate medicines free of cost for 15 days. The participants were explained the further course of action when there was no relief or if chronic treatment was deemed necessary.

### Statistical methods

The prevalence rates of various clinical conditions were estimated by calculating sex- and age-specific prevalence rates from the respondent population and applying these rates to the non-respondent population to obtain rates for the entire adult population. 95% confidence intervals (CI) were calculated for these rates. Student’s *t* test was used for comparison of means and Chi square test to compare proportions. Analyses were conducted using Stata 10.0 (Stata Corp, College Station, Texas, USA).

### Ethical approval

The research followed the tenets of the Declaration of Helsinki. Ethical approval for this nested study was granted as part of the main study, by the Institutional Ethical Committee of SEARCH formed according to the guidelines by the Indian Council for Medical Research. Consent was obtained first at the cluster level in the study villages 15 days before starting the survey. The community leaders (Village Council Leaders and members, school teacher and presidents of microfinance self help groups) were explained the purpose and scope of the study including the benefits to the villagers (availability of referral care in SEARCH clinic and the care through a village clinic). Informed written consent in vernacular language in a standard format was obtained from individual participants after explaining the nature and benefits of the study. The benefits provided during the study included free consultation by spine surgeons and rheumatologists in a clinic conducted in the same village at a later date. For those who needed further evaluation, laboratory investigations, as well as imaging with Magnetic Resonance Imaging (MRI) and x-ray including transport were provided free of cost. For patients needing pharmacotherapy, and physiotherapy, these services were also provided free of cost and for those needing surgical interventions, such services were provided at significantly subsidized costs. The CHW discussed these benefits using a printed pamphlet.

## RESULTS

**Figure 1:**
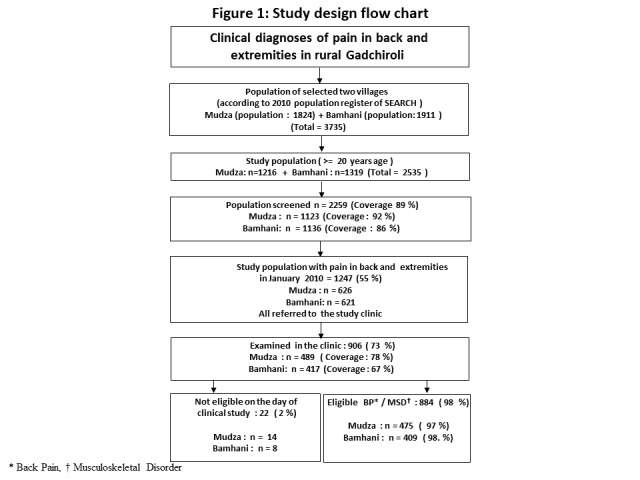
Study design flowchart. *Back pain. †Musculoskeletal disorder.

At the time of the study the combined population of both the villages was 3735. Out of these 2535 were ≥20 years of age and were eligible for the initial screening by the CHWs (phase 1). 2259 (89%) eligible adults were screened by CHWs and out of these 1247 (55%) adults had PBE. Of these, 906 (73%) attended the village clinic. Twenty-two participants (2%) were symptom free on the day of the clinical study and were excluded. The study flow is shown in **Figure 1**. The demographic characteristics of the participants and the non-participants are shown in **Table 1**. The non-participant group had higher number of younger, literate, labourer and male individuals compared to participants.

**Table 1:** Demographic characteristics of participants and non-participants in the study

Characteristics	Participants (n = 884)	Non participants (n = 363)	*P-*value
**Mean age (SD)**	48.5 (15.3)	43.1 (14.4)	<0.001
**Education, n (%):**			
Illiterate	464 (52)	163 (45)	0.0148
**Females**	558 (63)	194 (53)	0.0017
**Married**	845 (96)	340 (94)	0.1557
**Caste, n (%):**			
Scheduled caste	85 (10)	37 (10)	0.7552
Scheduled tribe	129 (15)	51 (14)	0.8042
Others	670 (76)	275 (76)	0.9898
**Occupation, n (%):**			
Labour	351 (40)	171 (47)	0.016
Farmer	309 (35)	133 (37)	0.5722
Service	22 (2)	11 (3)	0.5883
Household work	98 (11)	17 (5)	<0.001
Business	61 (7)	23 (6)	0.7180
Other	43 (5)	8 (2)	0.031

SD – standard deviation

The overall point prevalence of back pain in the adult population was 49%. Non -specific low back pain formed the largest group with a point prevalence of 45% in the population followed by non-specific neck and non-specific upper back pains (prevalence of 21% and 10% respectively) (**Table 2**). The point prevalence of radiculopathy was 12%, myelopathy was 0.4%, spinal tuberculosis was 0.1%, and spinal fracture was 0.1% (**Table 2**).

**Table 2:** Clinical patterns and their prevalence in the community among adults with back pain

Clinical patterns*	Number of patients in the clinic	proportion in the clinic (n = 884) %	Estimated point prevalence among adults† % (95% CI)
Nonspecific low back pain	728	82	45 (43.4, 47.5)
Nonspecific neck pain	330	37	21 (18.9,22.4)
Nonspecific mid back pain	166	19	10 (9.1, 11.7)
Myelopathy	6	1	0.4 (0.1,0.7)
Radiculopathy	187	21	12 (10.4,13.1)
Serious spinal pathologies:‡	3	0.3	0.2 (0.048, 0.45)
Tuberculosis	1	0.1	0.1 (0.001, 0.24)
Fracture	2	0.2	0.1 (0.01,0.31)

CI – confidence interval

*Categories are multiple and not exclusive.

†Adjusted for non-participants.

‡Suspected in the clinic based on prior radiological investigations available with the patients.

**Figure 2:**
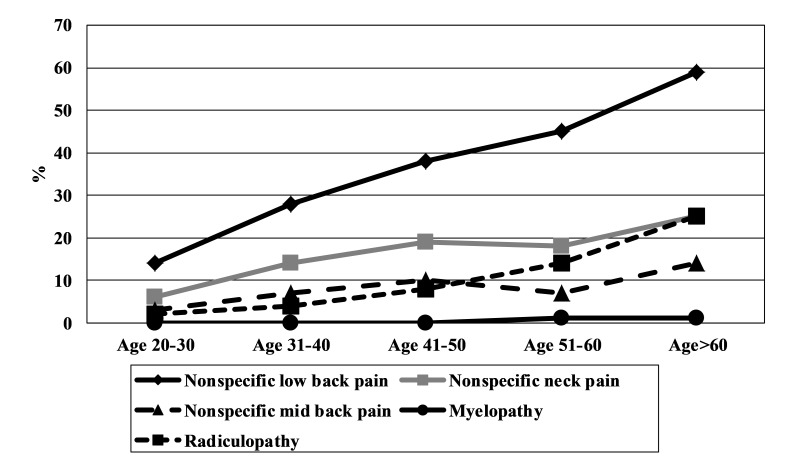
Age-wise population prevalence of clinical patterns of back pain.

The prevalence of non-specific low back pain increased with advancing age (**Figure 2**). Radiculopathy was common after the fifth decade of life. A higher prevalence among women than men was seen for non-specific low back pain (55.7% vs 34.1%, *P* < 0.001), non-specific neck pain (28.1% vs 12.1%, *P* < 0.001) and non-specific mid-back pain (13.6% vs 6.8%, *P* < 0.001) and radiculopathy (14.6% vs 7.3%, *P* < 0.001) (**Table 3**).

**Table 3:** Sex-wise estimated point prevalence of clinical patterns among adults with back pain

Clinical patterns*	Men† % (95% CI)	Women† % (95% CI)	*P*-value
Non-specific low back pain	34.1 (31.3, 36.9)	55.7 (52.8,58.6)	<0.001
Non-specific neck pain	12.1 (10.2, 14.2)	28.1 (25.5, 30.8)	<0.001
Non-specific mid back pain	6.8 (5.4, 8.5)	13.6 (11.7, 15.7)	<0.001
Myelopathy	0.3 (0.05, 0.8)	0.4 (0.14, 1.0)	0.52
Radiculopathy	7.3 (5.8, 8.9)	14.6 (12.6, 16.8)	<0.001
Serious spinal pathologies	0.3 (0.09, 0.92)	0.1 (0.002, 0.48)	0.16
Tuberculosis	0.0 (0, 0.33)	0.1 (0.002,0.48)	0.32
Fracture	0.3 (0.09, 0.92)	0.0 (0, 0.32)	0.04

CI – confidence interval

*Categories are multiple and not exclusive.

†Adjusted for non-participants.

## DISCUSSION

Among those with back pain, half of the adults had non-specific back pain, about quarter had non-specific neck pain and one in eight individuals had radiculopathy. Serious disorders such as myelopathy, tuberculosis of spine and spinal fractures were rare. The prevalence of non-specific back and neck pain was almost two times higher among women compared to men. The prevalence of non-specific back pain increased with age.

The prevalence of back pain in our study is higher than that reported from rural India or rural regions of Asia and South America [8]. The higher prevalence in the present study could be due to the predominant agrarian nature of the population where most of the individuals were farmers and involved in heavy physical labour. A study done in China had shown higher risk of low back pain among those involved in activities with heavy physical stress compared to those with mild physical stress [9]. In addition, lack of access to formal health care in this area could be a factor responsible for higher prevalence of back pain.

Among those with non-specific back pain the highest prevalence was seen for low back (45%) followed by neck (21%) and mid-back pain (10%). The pattern of pain being most common in the lower back followed by neck and mid-back was similar to those reported from other studies in rural India as well as other parts of the world [10].

The prevalence of non-specific back pain as a whole and at individual sites was almost two times higher among women compared to men. These findings are in agreement with previously published studies which have shown the prevalence of back pain to be higher among women [11,12].

Very little is known about the prevalence of spinal and neurological disorders other than non-specific back pain among those with PBE. In our study the prevalence of radiculopathy among adults was 12% (95% CI = 10.4%-13.1%). The prevalence of myelopathy, spinal tuberculosis and spinal fractures was low at 0.4%, 01% and 0.1% respectively.

Given the high prevalence of back pain and other spinal disorders which are associated with significant disability [3] and health care costs [13], a health care workforce needs to be created to treat disorders in rural areas. Since the prevalence of back pain was high and most of the back pain in the population was non-specific, village-level Accredited Social Health Activists (ASHAs) in the public health system in India or other community health workers can potentially provide care for this problem. These workers can be trained to refer those with red flags (**Box 1**) and treat patients with non-specific pain at the village level. Use of community health workers could be an important strategy to reduce the burden of back pain in the community. Patients with radiculopathy can be managed by the physicians at the primary health centers while those with myelopathy and other serious spinal disorders will need to be seen by internists or orthopedic surgeons at the district level. Patients needing spine surgeries need services of the spine surgeons or neurosurgeons who typically practice in large city centres.

Box 1 **Red flags in patients with low back pain.**

**History:**

1. Bladder/ bowel incontinence.
2. Peri-anal anesthesia.
3. Pain at night/ rest pain or constant pain.
4. Claudication in the lower limbs.
5. Thoracic pain.
6. Weight loss (subjectively perceived by the patient).
7. Fever (of at least 15 days in the recent past).
8. Past history of tuberculosis or cancer.
9. Difficulty in walking due to trauma– unstable gait, imbalance, tendency to fall, or requires support.
10. Deformity – coronal or sagittal, frontal or sideways.
11. Things falling from hand.
12. Tingling and / or numbness in hands.

**Examination:**

1. Toe or heel walking not possible due to weakness.
2. Weak hand grip and pinch.
3. Difficulty in bending (anyways).
4. Positive straight leg raising (SLR) test with pain on raising legs at less than 30°.

The study has several strengths such as being a community-based study from rural area of one of the underdeveloped districts of India with coverage close to 90% in the screening survey and the use of standardized questionnaires. The clinical evaluation was done by a team of clinical experts. There are also some limitations. The diagnosis was largely based on clinical examination. To mitigate this limitation, we used standardized case definitions and the team of clinicians was trained in a workshop so that same diagnostic criteria are used. Conducting community-based studies in resource limited settings remains challenging and use of standard clinical definitions is a common and accepted practice in large community-based prevalence studies. Furthermore, among those referred to the clinic, only about 70% actually attended the clinic which could result in a bias in the estimation of the point prevalence. To mitigate this limitation to some extent, we calculated sex- and age-specific prevalence among respondents and applied these rates to the non-respondent population to obtain prevalence in the entire adult population. Also, those without symptoms of back pain at the time of the initial screening survey could have been missed. This is likely to result in underestimation of the prevalence of back pain.

## CONCLUSION

Our study, conducted in one of the poorest communities of India, shows that among those with pain in the back and extremities, almost one in two individuals had non-specific back or neck pain. Radiculopathy is present in one in eight individuals. Serious spinal disorders are rare. Female sex and advancing age were associated with increasing prevalence of these disorders. The health care providers working in rural areas of India need to be trained to manage these disorders, given the high prevalence of back pain and provisions need to be made to provide care for this problem through the public health care system.
